# Supplementary material for: Patterns of Intron Gain and Loss in Fungi
Source: PLoS Biol. 2004 Nov 30;2(12):e422. doi: 10.1371/journal.pbio.0020422 (PMC532390; doi:10.1371/journal.pbio.0020422)
Supplement: Table S1 — Also available at http://genes.mit.edu/NielsenEtAl/. (4.3 MB ZIP). [file pbio.0020422.st001.zip › NielsenEtAl/html/1341.html]

AN4664.1.NCU09982.1.MG03075.1.FG09418.1


```
 CLUSTAL W (1.82) Multiple Sequence Alignments - Introns Inserted


Sequence 1: NCU09982.1	531 aa
Sequence 2: FG09418.1	512 aa
Sequence 3: MG03075.1	561 aa
Sequence 4: AN4664.1	522 aa
Alignment Length: 596 aa
Number Identitical Residues: 162 aa
Alignment Score (without introns) 9069


MG03075.1 	MAANTNRFSTYTTASADS--KDGE-KKKDMWNSMLESVASGKRLPEKNLLVL--------
NCU09982.1	MAANTNRYSTYTTASTGSDSRNGEKEKKELWSSMLDSVASAKRLPEKNILLL--------
FG09418.1 	---MANRVSVYTTGSGDSEGRDGE-QKKDLWTSMLESVASGKRLPEKNLIVL--------
AN4664.1  	-MSTSKRSSTVSS-------KDGL--KKNIWSSMLDSAATGKRLPEKNLLILGATALLSF
          	  : ::* *. ::       ::*   **::*.***:*.*:.*******:::*.:::  : 

MG03075.1 	---------G1GTVETQRSFLETLS-SDFRRTLDT------DHIPPVANHFALGNTYYDV
NCU09982.1	---------G~GSVDSQREFFESLSRNELRRNTDRQG----SRKPPIANSFALGYTYYDV
FG09418.1 	---------G1GSPEHQRDFLESLSSSEPRRNADR------LKIPPIANNFALGYTYYDV
AN4664.1  	GSSLTPPCLG~GTPESQREFLEAYSADTLDSSLSNEKRKGKGKVPPVANQFALGYTYLDV
          	.:: :... * *: : **.*:*: * .    . . .  ... : **:** **** ** **

MG03075.1 	LDADQED1ILARITLYSLTKPSASFSSLLKPLLTPQTIPNTLVVILLDWSHPWLWMRQLR
NCU09982.1	LDADQED~TLARISLYTLTDPSPAFASLIQPLITPQSIPNTLIVILLDWSQPWKWMRQLR
FG09418.1 	LDADQDD1TLARVSLYFLSQASTEFASLVAPLLTPETIPNTSLIILLDWSQPHLWLRQIW
AN4664.1  	LDADQED1TLARVSAYLLSEPSLSFAPLLKPLLTPQSIPETSVVILLDWSDPWTWVRRLR
          	*****:*  ***:: * *:..*  *:.*: **:**::**:* ::******.*  *:*:: 

MG03075.1 	DWILLLRTVLVSLSIEAKDAMEEVMIGWRDRGRGGASTNLDGSGVATAAMASDGGAGSDS
NCU09982.1	EWILLLRTVFQKLNRECSATMEEVMTVWRERGRGGGLN-LDGTSAVPTTSA-DA-----D
FG09418.1 	TWVQVIQEVLKKVDTDQHALMEEVMSAWKERGRGGAATNLDGTPSATATGS-DG-----D
AN4664.1  	EWVRLLRHVLVSLDDETKVVMEENMTQWRERKKG-----IDTSSTGSQATS--------S
          	 *: ::: *: .:. :    *** *  *::* :*     :* :   . : :        .

MG03075.1 	VALPLGPGEWEDALGLPLSVVCQN0ADKIDSLEKRQGWKEEQFDVVMQFMRTVLLKH1GA
NCU09982.1	VSLPVGPGEWEDALGMPLCVVCQN0AEKMDHLEKTQGWKEEEFDVVLQFLRTILLR-~--
FG09418.1 	SSLPLGPGEWEEPLGLPLCVVCQN0-----------AWKEPDFDTVLQYLRTVLLRH1GA
AN4664.1  	GSAIIGPGEWDEGLGVPLCVVCQG0TDKMEKLERDHGWSEDEFDFIHQFMRTLLVKH1GA
          	 :  :*****:: **:**.****. :.. .  .   .*.* :** : *::**:*::  .:

MG03075.1 	SLIYTSTSVPSQLPSLI~HSSLGITSLLKRQPLKHNVIDRDKIVVPPNWDSWGKIRVLRD
NCU09982.1	----------------L1HSSLGIHSLLKKQPLKHNVIDRDKILVPPNWDSWGKIRVLRE
FG09418.1 	SLIYTSQNAPSQLPSLI~HSTLGITSLLKRHPLKHNVIDRDKIVVPTNWDSWGKIRVLGG
AN4664.1  	SLIYTKPFHANNLHSVL~HSSLGIHSLLKKLSLKANFIERDKILIPTNWDSWAKIRVIRE
          	:   :.   ...  : : **:*** ****: .** *.*:****::*.*****.****:  

MG03075.1 	GFDVEMVSEGWSIDLDQPFPRESARTLNGDGLNEAG------EFSDSESDDGARQSEDDQ
NCU09982.1	GFDVEAVSSGWTADLSIPWPRQPQRVANGDDEQQGETNDTETGDEEHELDEDEYE-EPTE
FG09418.1 	TFDAEQVSNSWSEDIST--PRESMRFN---DDDQG------------EVDEAELE-EREQ
AN4664.1  	GSNLEGISTAWSIEIQD--PPEPLSDRN--DEAQ---------------DDGSSE-EDDG
          	  : * :* .*: ::.   * :.    .  .  :               *:   : *   

MG03075.1 	VEGSAVVLYEQKIENPSAAAQQHAALEDRSGRRMEVDTEDHQAFLEKQYPVLDEFRKTLQ
NCU09982.1	PDGSSVALYEASVQDPTMDALHIAGSSSHSTK-LEVQTTETQQFLEKQLKLLDVYK---Q
FG09418.1 	K--SAIARYEGWCRDPNSGGLAVVENAMHGEKWISVESDDTQDFLEKQLKILEAFK---A
AN4664.1  	T--SAVKIYEQTIKDPKRNTSLSYPGSQQSGNKIEVETLDMQGFLTKQLEVLEQLK----
          	   *::  **   .:*.           :. . :.*:: : * ** **  :*:  :    

MG03075.1 	KEEDEEAATKSKRSQLQGDSSASRGYGGSHGHDDYGHSPIGGLLGDQIGPVQFNMGGIQM
NCU09982.1	KNEEPTGHLARIKSARKTTSDS---LTAEDDHLKQQAEAK---VLEHIGPVQFNMGGIQV
FG09418.1 	KQPE------------KGSDNA---IARSTRHIDYSADEKS--ISEHIGPVQFNMGGIQV
AN4664.1  	---------------IEDEKDR---AAKKTPQLEMSPLEDNSRVNEHIGPVQFNMGGIQV
          	                :  ..       .  : .      .. : ::************:

MG03075.1 	DTDAAFQSIQ~RLT0ERQHAHEPEPEPDVDVEVDEGP-TDPDRMQAFFAGLIQRGMAKSG
NCU09982.1	DADDMLQRLK0ERQ~AFGTSP--EPLSPEDETVEETPQMDTENLQAFFTGLMNRSRQS--
FG09418.1 	DADDMLQRLK0DRN~AFSSSPSNEEEEAESPAANMAKDFDNEQLQSFFTGLMNRKGTADA
AN4664.1  	DADDMLQKLK0DRE~ASRSQRKDPVSSPGDEKA------NNQALANFFAGLVKKPGGSPR
          	*:*  :* ::           .       .  .      : : :  **:**:::      

MG03075.1 	GT-
NCU09982.1	---
FG09418.1 	SRS
AN4664.1  	GA-
          	.
```
